# The Position of St. George's Hospital

**Published:** 1920-07-24

**Authors:** 

**Affiliations:** *Treasurer.* St. George's Hospital, S.W. 1


### THE EDITOR'S LETTER-BOX.

**Correspondence on all subjects is invited, but we cannot in any way be responsible for the opinions expressed by our correspondents, who must give their name and address as a guarantee of good faith, but not necessarily for publication. Correspondents are reminded that brevity of style and conciseness of statement greatly facilitate early insertion.**

### The Position of St. George's Hospital.

*To the Editor of* THE HOSPITAL.

SIR,—In view of a certain amount of misconception on the part of the public as to the position of St. George's Hospital, I should like to state a few facts on the matter. Certain private negotiations (not of our seeking) were entered upon last year in the matter of a suggested amalgamation with another hospital, but such negotiations fell through because the proposer of the scheme found it impossible to obtain the necessary money for the purchase of the site of St. George's with a view to its being utilised for other public and charitable purposes in which he was interested.

It has been stated at various times that St. George's is "sitting" on a valuable site, but although the site was up for sale for a whole year some years ago, no adequate offer was received. I should further like to point out that there are various restrictions and conditions attached to about half of the site which very greatly reduce its saleable value, and the governors have definitely decided at the earliest opportunity to extend and improve, and eventually rebuild the hospital upon its present site at Hyde Park Corner. It is hoped that this statement will remove any doubt in the minds of the general public that the hospital is to be moved.—Yours faithfully,

GREVILLE, *Treasurer.*

St. George's Hospital, S.W. 1,
July 21, 1920.

